# 205 Hidden benefits in non-effective interventions: A subgroup analysis of associations between characteristics of study participants and changes in prolonged sedentary time using compositional data analysis

**DOI:** 10.1093/eurpub/ckae114.075

**Published:** 2024-09-26

**Authors:** Lisa Voigt, Antje Ullrich, Fabian Kleinke, Neeltje van den Berg, Marcus Doerr, Sabina Ulbricht

**Affiliations:** University Medicine Greifswald, Internal Medicine B, Greifswald, Germany; German Centre for Cardiovascular Research (DZHK), partner site Greifswald, Greifswald, Germany; German Centre for Cardiovascular Research (DZHK), partner site Greifswald, Greifswald, Germany; University Medicine Greifswald, Institute for Community Medicine, Department of Prevention Research and Social Medicine, Greifswald, Germany; German Centre for Cardiovascular Research (DZHK), partner site Greifswald, Greifswald, Germany; University Medicine Greifswald, Institute for Community Medicine, Section Epidemiology of Health Care and Community Health, Greifswald, Germany; German Centre for Cardiovascular Research (DZHK), partner site Greifswald, Greifswald, Germany; University Medicine Greifswald, Institute for Community Medicine, Section Epidemiology of Health Care and Community Health, Greifswald, Germany; University Medicine Greifswald, Internal Medicine B, Greifswald, Germany; German Centre for Cardiovascular Research (DZHK), partner site Greifswald, Greifswald, Germany; German Centre for Cardiovascular Research (DZHK), partner site Greifswald, Greifswald, Germany; University Medicine Greifswald, Institute for Community Medicine, Department of Prevention Research and Social Medicine, Greifswald, Germany

## Abstract

**Purpose:**

Interventions aiming to increase physical activity (PA) or reduce sedentary time (ST) often suffer from selection bias of highly active individuals. Subgroup analysis among most sedentary participants may reveal information about beneficial behaviour changes beyond non-significant intervention effects. We aimed to identify characteristics of participants of a non-effective intervention study associated with a reduction of time spent in prolonged sedentary bouts using compositional data analysis (CoDA) in a subsample of those with highest baseline bout levels.

**Methods:**

Data was used from an intervention study aiming to increase PA and reduce ST via brief feedback letters among adults from the general population aged ≥65 years. Participants wore ActiGraph-accelerometers on the hip during waking hours for seven days at baseline and 12-months follow-up (N = 130). We conducted a subgroup analysis among participants of the upper half of the distribution of ST spent in > 30min-bouts (i.e., median split at 2.6h/day; n = 69). CoDA was used to build a z1 coordinate representing the change in ST spent in > 30min-bouts, relative to the change of time spent in PA and in ≤ 30min-ST-bouts. Quantile regressions were used to investigate associations of age, sex, partnership, baseline waist circumference (WC), and baseline >30min-ST-bouts with z1 on percentiles 25, 50, and 75 of the z1 distribution.

**Results:**

Participants were aged 72±5.1 years, 61% were female, 74% lived with a partner, and baseline WC was 91±12.8 cm in women and 101±11.6 cm in men. At baseline (and follow-up), individuals spent 26% (25%) of time in > 30min-ST-bouts, 37% (38%) in PA, and 37% (37%) in ≤ 30min-ST-bouts. At the 25th percentile, female sex and higher WC were predictive for a higher reduction of > 30min-ST-bouts (ps<.05).

**Conclusions:**

In a non-effective intervention, among those with highest baseline levels (>2.6h/day) and highest reduction of prolonged ST (25th percentile), females and those with higher baseline WC reduced prolonged ST more than males and those with lower baseline WC. Intervention studies without significant efficacy may yield valuable information about individuals who showed beneficial behaviour changes.

**Funding Source:**

Federal Ministry of Education and Research (BMBF) as a site project of the German Centre for Cardiovascular Research (no. 81Z7400174)

